# Editorial: Primary and secondary hyperparathyroidism: from etiology to treatment

**DOI:** 10.3389/fendo.2025.1694239

**Published:** 2025-11-19

**Authors:** Pier Francesco Alesina, Gianluca Donatini, Fabio Medas

**Affiliations:** 1 Helios Klinikum Wuppertal, University of Witten-Herdecke, Wuppertal, Germany; 2 Universite de Poitiers, Poitiers, France; 3 Universita degli Studi di Cagliari, Cagliari, Italy

**Keywords:** parathyroid - adenoma, hyperparathyroidism, primary hyper parathyroidism, secondary hyperparathyroidism, parathyroidectomy

Primary and secondary hyperparathyroidism (HPT) remain challenging and multifaceted conditions at the crossroads of endocrinology, surgery, nephrology, and pathology (1, 2). Although significant progress has been made in diagnostic and therapeutic strategies, many aspects of these disorders are still under discussion, including surgical indications, imaging modalities, and the role of newer, minimally invasive therapies. The aim of this Research Topic was to provide a platform for clinicians and researchers to present novel findings, share clinical experiences, and stimulate debate regarding all aspects of HPT — from etiology and diagnosis to surgical and non-surgical treatment strategies. Contributions to this Topic reflect the broad spectrum of HPT and highlight the interdisciplinary nature of its management. The articles cover primary HPT, secondary HPT (mainly associated with chronic kidney disease), rare presentations, and emerging techniques. A mini-review and case report by Meomartino et al. illustrates the diagnostic challenges of intrathyroidal parathyroid adenomas, particularly when misclassified as indeterminate thyroid nodules. The authors highlight the potential value of preoperative calcium-phosphate screening in such cases to avoid unnecessary or incorrect interventions. The study by Hargitai et al. questions whether intraoperative PTH monitoring is still necessary in patients with concordant preoperative imaging undergoing minimally invasive parathyroidectomy. Their findings demonstrate a significant increase in persistent disease when IOPTH is omitted, supporting its continued use even in apparently straightforward cases. Zhou et al. present a large retrospective study evaluating over 700 patients with secondary hyperparathyroidism. Their findings emphasize the importance of anatomical and functional imaging—specifically the combination of 99mTc-MIBI SPECT/CT and high-frequency ultrasound—for accurate preoperative localization, particularly given the high incidence of ectopic glands. Li et al. compare microwave ablation with surgical parathyroidectomy, concluding that both improve bone mineral density and metabolic parameters, although surgery appears more effective at reducing PTH levels. Microwave ablation, however, shows fewer complications, suggesting a potential role in a well selected group of patients. A rare and instructive case by Dinoi et al. discusses the difficulty of distinguishing between brown tumors and bone metastases in the context of parathyroid carcinoma. The case underscores the clinical relevance of this distinction, as management and prognosis differ substantially. The authors advocate for histological confirmation, even in cases of high clinical suspicion. The metabolomic study by Gan et al. offers a novel perspective on SHPT in non-dialysis CKD patients. Their untargeted metabolomic profiling reveals significant differences in amino acid and lipid metabolism, suggesting potential biomarkers and therapeutic targets for early intervention. Lastly, Wang et al. address the role of parathyroid autotransplantation in preventing permanent hypoparathyroidism following total thyroidectomy with bilateral central neck dissection. Their findings suggest that while the risk of transient hypoparathyroidism increases with the number of autotransplanted glands, permanent hypoparathyroidism was observed only in patients who did not undergo autotransplantation. The articles selected for this Research Topic provide a comprehensive snapshot of the current landscape in hyperparathyroidism research. They emphasize the need for precise diagnosis, individualized treatment, and continued exploration of new diagnostic and therapeutic tools. The Topic also reflects the increasing interest in minimally invasive alternatives and the integration of molecular techniques into clinical practice. We thank all authors and reviewers for their valuable contributions. We hope that this Research Topic will inspire further research and collaboration to improve outcomes for patients affected by hyperparathyroid disorders.
